# Early Life Stress Enhances Cognitive Decline and Alters Synapse Function and Interneuron Numbers in Young Male APP/PS1 Mice

**DOI:** 10.3233/JAD-230727

**Published:** 2023-11-21

**Authors:** Niek Brosens, Dimitris Samouil, Sabine Stolker, Efthymia Vasilina Katsika, Sascha Weggen, Paul J. Lucassen, Harm J. Krugers

**Affiliations:** a Brain Plasticity Group, SILS-CNS, University of Amsterdam, Amsterdam, The Netherlands; b Department of Neuropathology, Heinrich-Heine-University, Düsseldorf, Germany

**Keywords:** Alzheimer’s disease, calretinin, cognitive flexibility, early life stress, glutamatergic proteins, synapses

## Abstract

**Background:**

Exposure to stress early in life increases the susceptibility to Alzheimer’s disease (AD) pathology in aged AD mouse models. So far, the underlying mechanisms have remained elusive.

**Objective:**

To investigate 1) effects of early life stress (ELS) on early functional signs that precede the advanced neuropathological changes, and 2) correlate synaptosomal protein content with cognition to identify neural correlates of AD.

**Methods:**

APPswe/PS1dE9 mice and littermates were subjected to ELS by housing dams and pups with limited bedding and nesting material from postnatal days 2-9. At 3 months of age, an age where no cognitive loss or amyloid-β (Aβ) pathology is typically reported in this model, we assessed hippocampal Aβ pathology, synaptic strength and synapse composition and interneuron populations. Moreover, cognitive flexibility was assessed and correlated with synaptosomal protein content.

**Results:**

While ELS did not affect Aβ pathology, it increased synaptic strength and decreased the number of calretinin^+^ interneurons in the hippocampal dentate gyrus. Both genotype and condition further affected the level of postsynaptic glutamatergic protein content. Finally, APP/PS1 mice were significantly impaired in cognitive flexibility at 3 months of age, and ELS exacerbated this impairment, but only at relatively high learning criteria.

**Conclusions:**

ELS reduced cognitive flexibility in young APP/PS1 mice and altered markers for synapse and network function. These findings at an early disease stage provide novel insights in AD etiology and in how ELS could increase AD susceptibility.

## INTRODUCTION

The etiology of Alzheimer’s disease (AD) is complex and multifactorial, encompassing a wide variety of risk and protective factors [1, 2]. Central in the early stage of the disease is the amyloid-β (Aβ) pathogenic pathway that is thought to trigger a cascade of deleterious events. In rare familial AD cases, genetic predisposition due to mutations in the amyloid precursor protein (*APP*) accounts for abundant and early presence of Aβ pathology and accelerated disease progression [3], whereas in the more prevalent sporadic variant, environmental and ‘lifestyle’ factors, like stress or diet, are regarded as important players in disease pathogenesis, next to agingargethispage3pt per se [4, 5]. Based on the genetic risk, environmental factors are suggested to shape vulnerability/resilience to AD, via modulation of brain and/or cognitive reserve, i.e., the ability of the brain to tolerate, compensate, and/or withstand effects of neurodegenerative pathology [6–9].

Stress is considered a relevant environmental risk factor for neurodegenerative disorders like AD [10–12]. Both preclinical and clinical studies support a role for stress in promoting the onset and progression of AD pathology [10, 13, 14]. Several possible mechanisms have been proposed, including accelerated aging, a primed genetic response to AD pathology, and an increase in the allostatic load, affecting the cognitive reserve [15–18]. In any case, the neurobiological effects of stress strongly overlap with AD-associated impairments, including synaptic and inhibitory network dysfunctions [19–21]. In fact, early stages of AD are accompanied by aberrant synapse loss and inhibitory network dysfunction [22, 23]. More specifically, loss of synapses and interneuron populations correlate with AD-associated cognitive decline and Aβ deposition, respectively [24, 25]. Additionally, several studies have now highlighted the potential role of the synaptic composition of glutamatergic proteins in susceptibility/resilience to Aβ pathology [26–28].

A particularly vulnerable time window for the effects of stress is the early postnatal period. This crucial developmental period determines the sensitivity to stress and cognitive function later in life [29, 30]. Previous research in our laboratory has shown that exposure to early life stress (ELS) leads to an increased susceptibility to AD pathology and cognitive decline in aged APPswe/PS1dE9 (APP/PS1) mice, a mouse model for Aβ pathology [13]. The mechanism that precedes this increased susceptibility to AD pathology following ELS has remained unclear, however.

Therefore, we here aimed to assess: 1) the effects of ELS on early signs of AD pathology, i.e., prior to the emergence of pathology at a later stage, and 2) neural correlates of ELS induced changes. To that end, APP/PS1 male mice were exposed to ELS and at 3 months of age, i.e., an age at which no cognitive loss or Aβ plaques are generally reported yet [31, 32], we studied hippocampal synaptic function and interneuron populations. We hypothesized that synaptic function and interneuron populations would be affected in 3-month-old APP/PS1 mice that were exposed to ELS. Moreover, we tested whether ELS enhanced cognitive impairments in APP/PS1 mice specifically, and if cognitive performance correlated with hippocampal synaptosomal protein content.

## MATERIALS AND METHODS

### Mice and breeding

All animal experiments were conducted under Dutch national law and in compliance with the European Union directive 2010/63/EU. The study design was evaluated and approved by the animal welfare committee of the University of Amsterdam. Mice were housed at a temperature of 20–22°C and a humidity of 40–60% with an *ad libitum* food availability (standard chow; 801722 CRM (P), Special Diets Services, Essex, UK) and water. Mice were kept on a 12 : 12 h light-dark cycle (lights on at 8 : 00 a.m., lights off at 8 : 00 p.m.) unless stated otherwise. For this study, 3-month-old wild type (WT) and APPswe/PS1dE9 (APP/PS1) male littermates were used [33]. Experimental animals were bred in house to standardize the perinatal environment. Two 6-week-old C57Bl/6J virgin WT females (Harlan Laboratories B.V., Venray, The Netherlands) and one bi-genic hemizygous APP/PS1 male were housed together one week for mating. Subsequently, females were pair-housed for a week in a clean standard cage with one nestlet (5×5 cm cotton nesting material: Tecnilab-BMI, Someren, the Netherlands) to practice nest making with the material. Hereafter, females were housed individually in a type II cage with nesting material and a filter top. Eighteen days after the start of the breeding, pregnant females were inspected daily before 9 : 00 a.m. for the birth of pups. Upon observation of a new litter, the previous day was designated as postnatal day 0 (P0).

### Early life stress

At P2, the limited bedding and nesting material model was applied to induce ELS until P9, as described previously [13, 34, 35]. Briefly, litters were culled to 6 pups per litter. Dams and corresponding litters were weighed and randomly assigned to the ELS or control (Ctrl) condition. Ctrl cages consisted of a standard amount of sawdust bedding material with one nestlet, whereas ELS cages consisted of a thin layer of sawdust bedding material covered with a fine-gauge stainless steel mesh placed at 1 cm above the cage floor, and only half a nestlet (2.5x5 cm). The ELS condition leads to fragmented and unpredictable maternal care from P2-P9 [29, 36, 37]. Cages of both conditions were covered with a filtertop and were left undisturbed. At P9, the dams and litters were weighed and placed in standard cages with standard amounts of bedding and nesting material. At P28, mice were weaned, and ear tissue was collected for identification and genotyping. Littermates were housed with 2–6 mice per cage. All animals were left undisturbed (except for weekly cage cleaning) until the experimental procedures at 3 months of age. As the effects of ELS are particularly sex specific [34], all experiments were further conducted with male mice. Separate cohorts were assigned for immunohistochemistry analysis, electrophysiology, and behavior.

### Genotyping

Ear clipped samples were used for DNA extraction with extraction (E7526; Sigma-Aldrich), tissue (T3073; Sigma-Aldrich) and neutralization solution B (N3910; Sigma-Aldrich) preparation solutions, according to manufacturer instructions. DNA extractions were used to perform standard PCR with a GoTaq G2 DNA polymerase kit (M7845; Promega) according to manufacturer instructions with the following primers (Eurogentic, SePop Desalted, 100μM in H_2_O, final primer concentration: 1μM): GACTGACCACTCGACCAGGTTCTG (forward), CTTGTAAGTTGGATTCTCATATCC (reverse). The PCR program was as follows: 95°C for 5 min, 35 cycles of 94°C for 45 s; 60°C for 45 s; 72°C for 1 min and 30 s, a final step of 72°C for 5 min. PCR products were run on a 2% agarose gel and imaged using a ChemiDoc Imaging system (Bio-Rad).

### Tissue preparation

Male mice were sacrificed by quick decapitation. The brains were removed, and the right hemisphere was immersion-fixed in 4% paraformaldehyde in phosphate buffer (0.1 M PB, pH 7.4) for 24 h at 4°C and then stored in 0.01% sodium-azide in 0.1 M PB at 4°C until further processing. Prior to tissue slicing, the fixed hemispheres were overnight cryoprotected in sequentially 15% and 30% sucrose in 0.1 M PB. Frozen tissue was cut in 40μm thick coronal sections in six parallel series using a sliding microtome. The slices were stored in antifreeze solution (30% ethylene glycol, 20% glycerol, 50% 0.05M PBS) at –20°C until immunohistochemical staining. From the left hemisphere, the hippocampus was isolated and stored at –80°C until western blot analysis for BACE1 content.

### DAB immunohistochemistry

Brain slices were washed 3×5 min in 0.01 M PB and then mounted onto pre-coated glass slides (Superfrost Plust slides, Menzel, Germany). The slides were dried overnight and were washed 3×5 min in 0.05 M tris-buffered saline (TBS) with 0.01% triton X-100 (TBST). Antigen retrieval was performed by boiling the sections in a microwave (±95°C) in 0.01M citrate buffer (pH 6) for 15 min. After cooling down at room temperature (RT), slides were washed for 5 min in TBST. A 30 min incubation step with 0.3% H_2_O_2_ was used to block endogenous peroxidase activity. Another blocking step to suppress background was performed for 30 min using 1% bovine serum albumin (BSA) with TBST. Hereafter, an incubation with the primary antibody (mouse anti-6E10, SIG-3932-1000, BioLegend) in 0.05M TBS with 1% BSA and TBST at a 1 : 1500 ratio was performed for 2 h at RT and overnight at 4°C. The slides were washed 3×5 min in TBST, followed by a 1 : 200 secondary antibody incubation with a biotinylated sheep anti-mouse antibody (GE healthcare) in 1% BSA TBST at RT for 2 h. Slides were washed 3×5 min in TBST. Subsequently, 1 : 800 ABC elite (Elite Vectastain Brunschwig Chemie, Amsterdam) was applied in TBST for 90 min. After washing 1×5 min in 0.05 M and 4×s5 min in 0.05 M TB, the tissue was finally incubated in 0.2 mg/mL diaminobenzidine (DAB) in 0.05 M TB with 0.01% H_2_O_2_ and 2 mg/ml nickel ammonium sulfate at a pH of 7.6 for 12 min. Slides were washed 3×5 min in 0.05 M TB and dehydrated using increasing concentrations of EtOH (50%, 75%, 96% and 100%, 2 min for each concentration) followed by xylol for 2 min. Finally, slides were coverslipped using Entellan (EMD Millipore, Billerica, MA, USA).

### Fluorescent immunohistochemistry

Free-floating sections were washed in 0.1 M phosphate-buffered saline (PBS) with 0.5% Triton X-100 (PBST). Sections were then incubated with Fab fragments (1 : 200, Affinipure Fab Fragment Goat Anti-Mouse IgG, Lot #145879, Jackson ImmunoResearch) in 0.1 M PBS for 30 min at RT. A washing step of 3×10 min in PBST was performed. Subsequently, blocking was performed with 10% normal goat serum (NGS) in PBST for 1 h at RT. A primary antibody mix was applied containing 1 : 1000 Rabbit anti-Calretinin (7699/3 H, SWANT) and 1 : 800 mouse anti-Parvalbumin (P3088, Sigma-Aldrich) in the blocking mix overnight at 4°C. After a 3×10 min wash with PBST, sections were treated with a secondary antibody mix containing 1 : 800 Alexa Fluor Donkey Anti-Rabbit 488 (10424752, Invitrogen) and 1 : 800 Alexa Fluor Goat Anti-Mouse 594 (A21125, Life Technologies, Invitrogen) in the blocking mix for 2 h at RT. Sections were washed 2×10 min in 0.1 M PBS followed by one final wash in 0.1 PB for 10 min. Sections were mounted on glass slides and Vectashield with DAPI (Vectashield Mounting Medium with DAPI, H-1200, Vector Laboratories Inc.) was used for cover slipping.

### Imaging and quantification

The 6E10 DAB staining was imaged using a Nikon DS-Ri2 microscope in a widefield setting at 10×magnification. Images were assigned to corresponding bregma points and ROIs were selected in the ventral (–1.22 mm to –2.18 mm) and dorsal (–2.3 mm to –3.4 mm) hippocampus (CA subregions and DG) and in the prefrontal cortex (layer II/III of the mOFC, lOFC, ACC, IL, PL) using ImageJ analysis software (version 1.52a, Wayne Rasband). Images were converted to 8-bit grey scale and the mean integrated density was calculated for each ROI in 5–7 coronal sections per animal. The corrected integrated density (CID) was calculated according to the following formula: integrated density ROI –(ROI surface x mean grey value of background).

The interneuron staining was imaged using a Nikon DS-Ri2 microscope in fluorescent setting at 10x magnification and hippocampal ROIs were selected based on the same bregma coordinates as the analysis of the 6e10 staining. Images were converted to 8-bit grey scale to visualize calretinin (CR) and parvalbumin (PV) positive cells. CR^+^ and PV^+^ cells were manually counted and the respective ROI areas were measured. Throughout the counting, a threshold of minimum mean grey value was set to deem whether a cell was positive or negative. Counted cells were then converted to the number of cells per mm^2^. Throughout analysis, the experimenters were unaware of the coding of the sections.

### Recording miniature excitatory postsynaptic currents (mEPSCs)

Mice were sacrificed by quick decapitation. The brains were rapidly removed and placed in ice-cold oxygenated (95% O_2_/5% CO_2_) artificial cerebrospinal fluid (ACSF: in mM): 118 NaCl, 2.5 KCl, 26 NaHCO_3_, 1 NaH_2_PO_4_, supplemented with 1 MgCl_2_, 2 CaCl_2_, 22 glucose. Coronal slices (350μm) were made in ice-cold slicing ACSF (sACSF) using a vibratome (VT1000 S, Leica). The sACSF contained (in mM): 139 C_5_H_14_ClNO, 3.5 KCl, 0.5 CaCl_2_, 6 MgSO_4_, 1.25 NaH_2_PO_4_, 25 NaHCO_3_, and 10 D-glucose, saturated with 95% O_2_–5% CO_2_. For recovery, slices were incubated for 20 minutes in warm (32°C) oxygenated ACSF followed by a 1-h incubation in oxygenated ACSF at RT. Individual slices containing the dorsal hippocampal area (Bregma –2.0 mm to –3.2 mm) were transferred into a recording chamber submerged in a constant flow of oxygenated ACSF with 1μM TTX (Tocris) and 100μM picrotoxin (Sigma). Whole cell recordings in current clamp were performed using a DIC microscope (Axioskop 2 FS Plus, Zeiss) with a water immersion 40×objective (0.8 W), equipped with a CCD Camera (TVCCD 624, Monacor) and a headstage (CV 203BU, Axon Instruments) assembled to a motorized micromanipulator (Scientific). For all the recordings, borosilicate glass pipettes (1.5 mm outer diameter, Hilgerberg, Malsfeld, Germany) were pulled with a Sutter (USA) micropipette puller to establish a pipette resistance of 3-6 m*Ω*. The pipette solution contained (in mM): 115 CsMeSO_3_, 20 CsCl, 10 HEPES, 2.5 MgCl_2_, 4 Na_2 - _ATP, 0.4 Na-GTP, 10 Na-Phosphocreatine, 0.6 EGTA. The pH of this intracellular solution was 7.2 (adjusted with KOH) and the osmolarity was 290.55 mOsm. Under positive pressure, the electrode was directed towards a neuron in the suprapyramidal blade of the dentate gyrus. Once a seal was established on the cell membrane (resistance > 1G*Ω*), the membrane patch was ruptured by gentle suction and kept at a holding potential of –65 mV. Neurons with access resistance < 30 M*Ω* were used for whole cell recordings in voltage clamp. Cells were recorded for 10 min and the recordings were amplified using a Axopatch 200B amplifier and digitized with a Axon Digidata 1550A. Data acquisition was performed in pClamp 10.7 and mEPSCs were analyzed with MiniAnalysis (Synaptosoft). Individual events above a 5 pA threshold were manually selected.

### Behavioral tests

A separate cohort of mice was bred to perform a multiple choice-reversal learning paradigm (MCRL) in automated home cages (Phenotyper; model 3000, Noldus Information Technology) in which behavior was tracked with videotracking software (Ethovision XT 11). Phenotyper protocols were obtained from Sylics (Synaptologics BV, the Netherlands). Throughout the behavioral experiment, mice were kept on an 8 : 00 a.m.–8 : 00 p.m. light-dark cycle. The Phenotypers were equipped with a triangular-shaped shelter with two entrances on one side and *ad libitum* access to a water bottle and a feeding station with standard chow. To provide a high contrast with dark-colored mice under infrared light, approximately 2–3 cm of white α-cellulose bedding was provided (ALPHA-Dri, Shepherd Specialty Papers Inc.). No bedding change was required throughout the experimental period. The mice were weighed and introduced to the Phenotyper cages 3 days before onset of the MCRL paradigm (during light phase; 14 : 00–16 : 00). Parameters of spontaneous behavior were assessed during the 3 days of habituation. Subsequently, the regular chow was taken away and the CognitionWall (operant wall with 3 gates) was placed in front of a pellet dispenser tube. Mice were habituated to the CognitionWall for 15 min before onset of the experiment. 5 free pellets (Dustless Precision Pellets, 14 mg, Bio-Serve) were delivered. Until mice entered one of the gates 10 times or more and entered the left gate at least 5 times, no food reward was dispensed. These entries were used to determine whether the mice had an initial preference or bias towards one of the three gates. Following the preference phase, discrimination learning (DiL) commenced for the next 2 days (DiL1 and DiL2). Mice earned their food by going through the left gate of the cognition wall. On day 3, reward probability was changed in favor of the right gate during reversal learning (RL) for two days (RL1 and RL2). For both DiL and RL, food rewards were delivered in a nonconsecutive FR5 (Fixed ratio) reinforcement schedule (every fifth correct entry was rewarded with one pellet) as a FR1 (every correct entry is rewarded with one pellet) reinforcement schedule leads to satiety [38]. Online monitoring of the experiments allowed evaluation of food intake during the phases. After RL completed, the mice were weighed and placed back in standard homecages until sacrifice.

### Synaptosome isolation

Three days after the mice were moved from the Phenotyper cages back to standard homecages, they were sacrificed by acute decapitation in the morning. Hippocampi were isolated and stored at –80°C until further use. Synaptosomal fractions were isolated as described in [39]. Briefly, hippocampi were homogenized in 5 mL of ice-cold iso-osmolar buffer containing 5 mM HEPES pH 7.4, 0.32 M sucrose and protease inhibitor cocktail EDTA free (04693132001, Roche) using a 7 mL glass Dounce homogenizer with 20×strokes of the pestle. The homogenate was centrifuged at 1000 ×g for 10 min at 4°C. The supernatant was layered on top of a 0.85/1.2 M sucrose density gradient and ultracentrifuged at 100,000 ×g for 2 h at 4°C. Synaptosomes were isolated from the 0.85/1.2 M sucrose interface, mixed with sample volume of 5 mM HEPES and iso-osmolar buffer to reach a final volume of 15 mL. The homogenate was centrifuged at 76,000 ×g for 30 min at 4°C. The pellet was resuspended in 500 mM HEPES and stored at –80°C until western blot analysis was performed.

### Western blots

The protein concentration of the samples was determined using a BCA Protein Assay (23225, Pierce (Thermo Fischer), The Netherlands). Samples containing 2-10μg protein in sample buffer were denaturized at 95°C for 5 min. Proteins were separated using an 8% polyacrylamide-SDS gel and transferred to a PVDF membranes (162-0177, Biorad, The Netherlands) using ProSieve EX transfer buffer (Lonza Bioscience, Allendale, USA). Blots were washed with 0.1 M TBS and 0.05% Tween 20 for 5 min followed by a blocking step with 5% milk in 0.1 M TBS and 0.05% Tween 20 for 2 h. The blots were incubated with primary antibodies overnight at 4°C in 0.1 M TBS with 5% BSA and 0.05% Tween 20. Primary antibodies included BACE1 (1 : 1000, D10E5, Cell Signaling, 70 kDa), PSD95 (1 : 5000, D27E11, Cell Signaling, 95 kDa), GluA1 (1 : 1000, 1504, Chemicon, 106 kDa), GluA3 (1 : 1000, 32-0400, Zymed, 99 kDa), GluN2A (1 : 1000, 5220, Chemicon, 180 kDa), GluN2B (1 : 1000, 5216, Chemicon, 175 kDa), α-tubulin (1 : 1000, 10D8, Santa Cruz) and GAPDH (1 : 1000, 2118 S, Bioke, 37 kDa). After washing with 0.1 M TBS with 5% BSA and 0.05% Tween 20, blots were incubated for 2 h at RT with corresponding antibodies (HRP conjugated, Biorad, The Netherlands). Blots were washed with 0.1 M TBS with 5% BSA and 0.05% Tween 20 and the bands were visualized by chemiluminescence using an ECL Prime kit (RPN2232, GE Healthcare, Amersham, GE, The Netherlands) and a Li-COR machine. Optical density was determined in ImageJ. Measurements of the proteins of interest were corrected for total protein (GAPDH or α-tubulin band).

### Statistical analysis

Data were analyzed using Rstudio and Graphpad Prism 9 and expressed as mean±standard error of the mean (SEM). Data were considered statistically significant when *p* < 0.05 (two-sided testing). Datapoints that were outside the 1.5x IQR range and had methodological or biological deviations were deemed as outliers and excluded from analysis. Assumptions for parametric analysis were tested using the Shapiro-Wilk normality test and the Levene’s test for homogeneity of variance. Subsequently, appropriate parametric or non-parametric statistic tests were performed. Greenhouse-Geisser correction was applied when the assumption of sphericity was violated. The data was nested, as multiple mice from the same litter were included in the experiments. Therefore, the contributing effects of litter to the outcome variable were tested and corrected where needed. The general approach was as follows: for Ctrl x ELS comparisons, independent t-tests (or non-parametric Wilcoxon rank-sum tests) were performed. For 2x2 comparisons of genotype x condition, a two-way ANOVA was performed with post-hoc Bonferroni testing. For the frequency and cumulative probability distributions of the mEPSC data, Anderson-Darling k-sample tests were performed to test differences in distributions. Mixed model ANOVA’s were used for behavioral data with repeated measures, where time or percentage were within-units and genotype and condition were between-units. Percentage criteria for the MCRL paradigm were assessed in a moving window of the last 30 entries (i.e., 80% criteria are reached when 24 of the last 30 entries are correct). To correlate cognitive performance with synaptosomal protein content, Pearson correlation tests were performed.

## RESULTS

Early life stress decreases bodyweight gain during early development

**Fig. 1:**
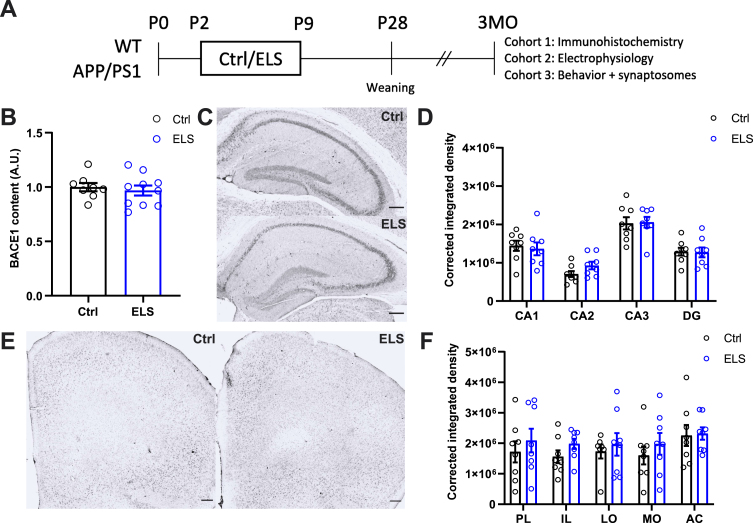
Early life stress does not affect amyloid pathology in the hippocampus and prefrontal cortex of 3-month-old APP/PS1 mice. A) Schematic timeline of the study. WT and APP/PS1 littermates were subjected to the ELS paradigm from postnatal day (p) 2-9. At P28, mice were weaned and earclipped for genotyping. Mice were left undisturbed until they were 3 months of age (3MO), at which cohort 1 was used for immunohistochemical analysis, cohort 2 for electrophysiological analysis, and cohort 3 for behavioral and synaptosomal protein content analysis. B) Hippocampal BACE1 content was not affected in APP/PS1 mice as a result of ELS (Ctrl *n* = 8, ELS *n* = 10). C) Representative images of hippocampal Aβ immunohistochemistry using 6E10 antibodies. D) No differences were observed between Ctrl and ELS APP/PS1 mice for corrected integrated density measurements of hippocampal subregions (Ctrl *n* = 8, ELS *n* = 8). E) Representative images of prefrontal cortex Aβ immunohistochemistry using 6E10 antibodies. F) No differences were observed between Ctrl and ELS APP/PS1 mice for corrected integrated density measurements of prefrontal cortex subregions (Ctrl *n* = 8, ELS *n* = 8). Data is presented as mean±SEM. Scale bar = 200μm.

WT and APP/PS1 mice were subjected to ELS by housing the dam and the litter with limited bedding and nesting material from P2-9 [13, 34, 35]. This resulted in a significantly lower body weight gain from P2-9 in the ELS group (T(38) = 4.36, *p* < 0.001) (Table 1), as reported previously [13, 34, 35]. Accordingly, the body weight gain from P9 until weaning at P28 normalized (T(38) = 1.64, *p* = 0.11) (Table 1). Hereafter, mice were genotyped and left undisturbed until the mice were 3 months of age (Fig. 1A). At this age, there were no differences in bodyweight anymore between the 4 experimental groups (Table 1).

**Table 1:** Early life stress and bodyweight gain. Early life stress decreased bodyweight gain in male mice from postnatal day (P) 2-9. From P9-P21, bodyweight gain normalized and at 3 months of age, there were no differences in bodyweights between conditions and genotypes.

	Ctrl		ELS	
P2-P9 BW gain	2.97±0.13 (20)		2.25±0.10 (20)***
P9-P28 BW gain	10.56±0.67 (20)		9.26±0.42 (20)
	WT	APP/PS1	WT	APP/PS1
3-month BW	30.17±0.51 (11)	29.60±0.81 (9)	29.16±0.92 (9)	29.42±0.55 (11)

Data is presented as mean±SEM. ***T-test compared to Ctrl mice, p < 0.001

### Early life stress does not affect amyloid pathology in the hippocampus and prefrontal cortex of 3-month-old APP/PS1 mice

At 3 months of age, we assessed the effects of ELS on hippocampal BACE1 levels and Aβ deposition in the hippocampus and prefrontal cortex. No differences between the 3-month-old Ctrl APP/PS1 and ELS APP/PS1 mice were present for hippocampal BACE1 levels as measured with a western blot analysis (T(16) = 0.51, *p* = 0.61) (Fig. 1B). Moreover, Aβ deposition was measured in subregions of the hippocampus and prefrontal cortex. Overall, no differences were found between Ctrl APP/PS1 and ELS APP/PS1 mice for the CA subregions and the DG (Fig. 1C, D). Similarly, no differences in Aβ deposition were found in the PL, IL, lORB, mORB, and ACC of the prefrontal cortex (Fig. 1E, F). These results show that ELS does not affect amyloid pathology in the hippocampus or prefrontal cortex of 3-month-old APP/PS1 mice.

### Early life stress decreases the CR^+^ interneuron population in the dentate gyrus of 3-month-old mice regardless of genotype

**Fig. 2:**
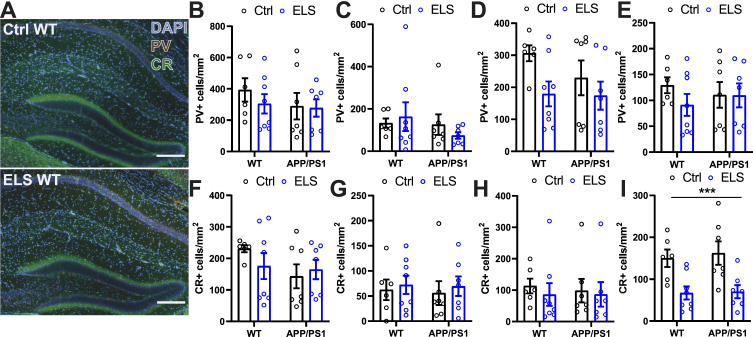
Early life stress decreases the CR^+^ interneuron population in the dentate gyrus of 3-month-old mice regardless of genotype. A) Representative images of hippocampal immunofluorescent parvalbumin and calretinin labelling. No differences were observed in the number of parvalbumin^+^ cells in the CA1 (B), CA2 (C), CA3 (D), and DG (E) between WT and APP/PS1 animals that were subjected to the ELS or Ctrl condition. No differences were observed in the number of calretinin^+^ cells in the CA1 (F), CA2 (G), CA3 (H) between WT and APP/PS1 animals that were subjected to the ELS or Ctrl condition. I) A significantly lower number of calretinin^+^ cells were observed in the DG as a result of ELS regardless of genotype (F_condition_(1,24) = 18.37, *p* = 0.0003). Data is presented as mean±SEM. Scale bar = 200μm. Ctrl WT *n* = 6, ELS WT *n* = 8, Ctrl APP/PS1 *n* = 7, ELS APP PS1 *n* = 7. ***two-way ANOVA main effect of condition *p* < 0.001.

To assess whether ELS affects the inhibitory network, we quantified the PV+and CR^+^ interneuron populations in the hippocampus and prefrontal cortex of 3-month-old WT and APP/PS1 mice. Regarding PV, no differences were found between the groups for the hippocampal CA subregions and the DG (Fig. 2B-E). For CR, no differences were found in the hippocampal CA subregions (Fig. 2F-H) whereas in the DG, a significantly lower number of CR+cells was observed after ELS (F_*condition*_(1,24) = 18.37, *p* = 0.0003) (Fig. 2I). These findings show that ELS leads to a selective decrease in the CR^+^ interneuron population of the hippocampal DG, while other subregions and the PV^+^ interneuron population remain unaffected at this age.

### Early life stress increases the mEPSC frequency of DG granular cells in 3-month-old mice regardless of genotype

**Fig. 3:**
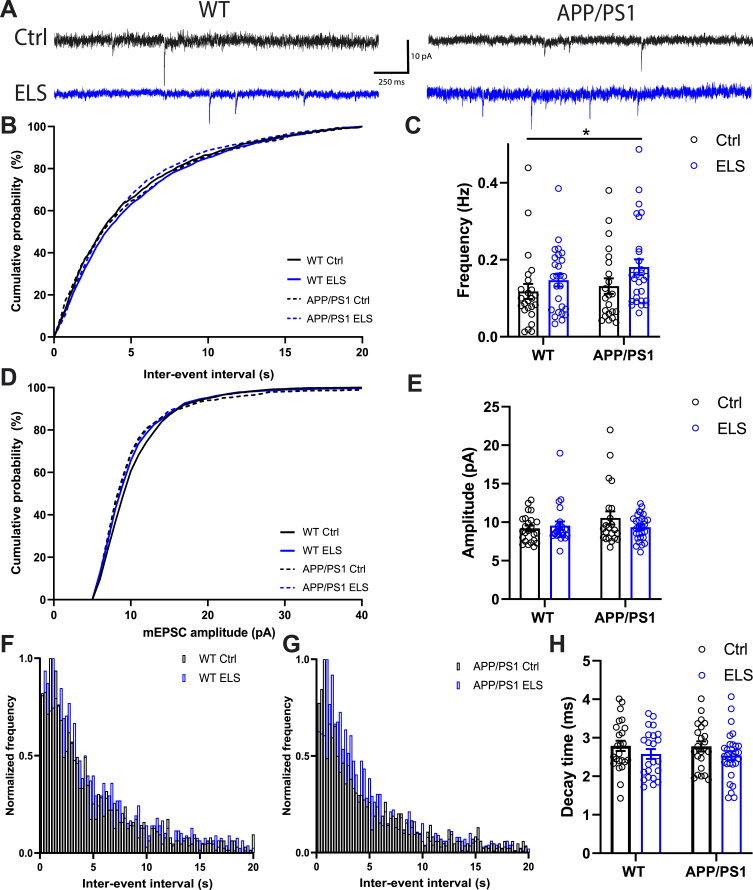
Early life stress increases the mEPSC frequency DG granular cells of 3-month-old mice regardless of genotype. A) Representative traces of mEPSCs recorded in DG granular cells. B) No differences were observed in the cumulative probability of the inter-event interval between WT and APP/PS1 animals that were subjected to the ELS or Ctrl condition. C) A significantly higher mEPSC frequency was observed as a result of ELS, regardless of genotype (F_condition_ (1,93) = 4.53, *p* = 0.04). D) No differences were observed in the cumulative probability of the mEPSC amplitude between WT and APP/PS1 animals that were subjected to the ELS or Ctrl condition. E) No differences were observed in the means of the mEPSC amplitude between WT and APP/PS1 animals that were subjected to the ELS or Ctrl condition. No differences were observed in normalized frequency of the inter-event interval between WT and APP/PS1 animals (F and G) that were subjected to the ELS or Ctrl condition. H) A slight trend towards a lower decay time was observed as a result of early life stress, regardless of genotype (F_condition_ (1,93) = 3.11, *p* = 0.08). Data is presented as mean±SEM. Ctrl WT *n* = 24 cells from 6 animals, ELS WT *n* = 26 cells from 6 animals, Ctrl APP/PS1 *n* = 22 cells from 6 animals, ELS APP/PS1 *n* = 28 cells from 6 animals. *two-way ANOVA main effect of condition *p* < 0.05.

To investigate whether ELS affects basal excitatory synaptic activity in the hippocampus of 3-month-old APP/PS1 mice, mEPSCs were recorded in granular cells of the DG. Overall, a significantly higher mEPSC frequency was present as a result of ELS, regardless of condition (F_condition_(1,93) = 4.53, *p* = 0.04) (Fig. 3C). No significant differences were found in the inter-event interval cumulative probability (Fig. 3B) and normalized frequency (Fig. 3F-G). In terms of amplitude, the means were similar between all groups as well as the cumulative probability (Fig. 3D-E). Finally, a slight trend towards a lower decay time as a result of early life stress was present, regardless of genotype (F_condition_ (1,93) = 3.11, *p* = 0.08) (Fig. 3H). These results show that ELS increases the mEPSC frequency regardless of genotype in the dentate gyrus of 3-month-old WT as well as APP/PS1 mice.

### 3-month-old APP/PS1 mice display enhanced locomotor activity compared to WT mice regardless of condition

**Fig. 4:**
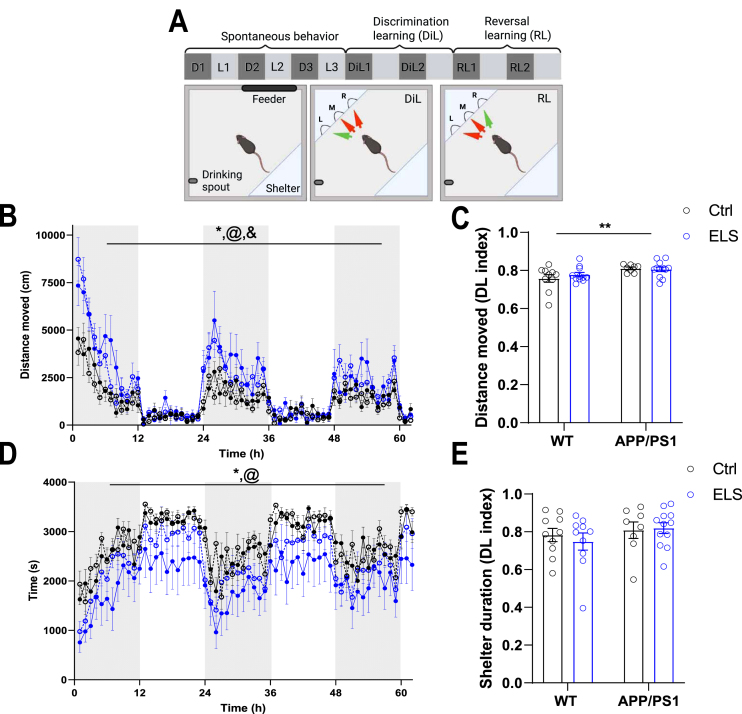
3-month-old APP/PS1 mice display enhanced locomotor activity compared to WT mice. A) Schematic overview of the behavioral paradigm in Phenotyper cages. Spontaneous behavior was assessed for the first 3 days (3 dark (D) and 3 light (L) phases) during habituation to the automated homecages. The 4^th^ day, discrimination learning (DiL) commenced for 2 days (DiL1 and DiL2) during which the left entrance is rewarding followed by 2 days of reversal learning (RL; RL1 and RL2) during which the right entrance is rewarding. Fig. was created with BioRender (https://app.biorender.com/). B) Distance moved in cm and in hourly bouts over the course of 3 days show a significant time effect (F_time_(61,2318) = 28.81, *p* < 0.001), a genotype effect (F_genotype_(1,38) = 21.8, *p* < 0.001) and a genotype x time interaction effect (F_genotypextime_(61,2318) = 3.47, *p* < 0.001). C) APP/PS1 mice have a significantly higher Dark/Light (DL) index for distance moved compared to WT mice (F_genotype_(1,37) = 9.98, *p* = 0.003). D) Time spend in the shelter in seconds and in hourly bouts over the course of 3 days reveals a genotype effect (F_genotype_(1,61) = 5.22, *p* = 0.03) and a time effect (F_time_(61,2318) = 18.81, *p* < 0.001). E) There are no differences between the groups in terms of the DL index for shelter time. WT Ctrl *n* = 10, WT ELS *n* = 10, APP/PS1 Ctrl *n* = 8, APP/PS1 ELS *n* = 12. Data is presented as mean±SEM. *genotype effect *p* < 0.05, ^@^time effect *p* < 0.001, ^&^genotype x time interaction effect *p* < 0.001.

To assess the effects of ELS on the behavior and cognition of 3-month-old APP/PS1 mice, mice were placed in Phenotyper homecages for one week (Fig. 4A). Mice were first habituated to the cages for 3 days, during which general behavioral readouts were assessed. Overall, the distance moved was significantly affected by time (F_time_(61,2318) = 28.81, *p* < 0.001) and APP/PS1 mice display a significantly higher distance moved compared to WT mice (F_genotype_(1,38) = 21.8, *p* < 0.001). Moreover, there was a strong interaction effect present for genotype and time (F_genotypextime_(61,2318) = 3.47, *p* < 0.001), as the APP/PS1 mice moved over larger distances during the dark phase specifically (Fig. 4B). To subsequently test whether APP/PS1 mice preferentially display higher locomotor activity during the dark phase, we calculated a Dark/Light (DL) index of the distance moved (dark phase/(dark + light phase). Using this index, APP/PS1 mice had significantly higher levels of distance moved compared to WT mice (F_genotype_(1,37) = 9.98, *p* = 0.003), indicating that APP/PS1 mice indeed travel larger distances specifically during the dark phase (Fig. 4C). In line with the enhanced locomotor activity, APP/PS1 mice also had spent less time in the shelter (F_genotype_(1,61) = 5.22, *p* = 0.03) and time influenced sheltering behavior (F_time_(61,2318) = 18.81, *p* < 0.001) (Fig. 4D). No significant differences in shelter time during the dark/light phases were present between the experimental groups (Fig. 4E).

### Early life stress significantly impairs reversal learning in 3-month-old APP/PS1 mice

**Fig. 5:**
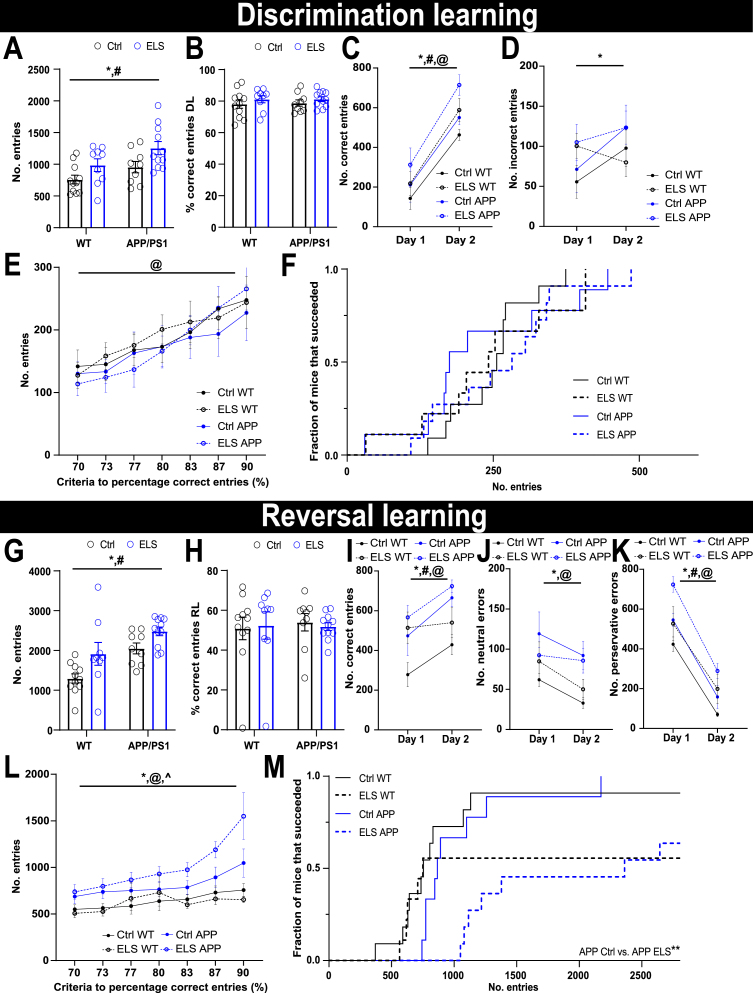
ELS impairs reversal learning in 3-month-old APP/PS1 mice. A) During DiL, APP/PS1 mice performed a higher number of total entries compared to WT mice (F_genotype_(1,36) = 6.45, *p* = 0.02) and ELS mice performed a higher number of total entries compared to Ctrl mice (F_condition_(1,36) = 8.24, *p* = 0.007). B) All groups displayed similar levels of percentage of correct entries. C) Split over 2 days, all animals increased the number of correct entries (F_day_(1,36) = 69.26, *p* < 0.001), APP/PS1 mice performed more correct entries compared to WT mice (F_genotype_(1,36) = 4.78, *p* = 0.04) and ELS mice performed more correct entries compared to Ctrl mice (F_condition_(1,36) = 7.32, *p* = 0.01). D) APP/PS1 mice performed more incorrect entries (middle and right entries) compared to WT mice (F_genotype_(1,36) = 5.89, *p* = 0.02). E) All groups similarly increased the number of entries required to reach the increasing criteria (F_*criteria*_(2.41,86.59) = 3.48, *p* < 0.001). F) All groups displayed a similar DiL succession rate at a criterion of 90% correct entries for individual mice in all experimental groups. G) APP/PS1 mice performed a higher number of total entries compared to WT mice (F_genotype_(1,36) = 15.59, *p* < 0.001) and ELS mice performed a higher number of total entries compared to Ctrl mice (F_condition_(1,36) = 9.75, *p* = 0.004). H) All groups displayed similar levels of percentage of correct entries during RL. I) Split over the 2 days of RL, all groups increase the number of correct entries (F_day_(1,36) = 22.58, *p* < 0.001). APP/PS1 mice perform more correct entries compared to WT mice (F_genotype_ (1,36) = 8.34, *p* = 0.007) and ELS mice perform more correct entries compared to Ctrl mice (F_condition_(1,36) = 4.66, *p* = 0.04). J) All groups decrease the number of neutral errors (middle entries) (F_day_(1,36) = 10.29, *p* = 0.003) and APP/PS1 overall perform more neutral errors compared to WT mice (F_genotype_(1,36) = 8.36, *p* = 0.006). K) All groups decrease the number of perservative errors (left entries) over the 2 days of RL days (F_day_(1,36) = 233.58, *p* < 0.001). L) Regardless of the 2 days, APP/PS1 mice perform more perservative errors compared to WT mice (F_genotype_ (1,36) = 8.38, *p* = 0.006) and ELS mice perform more perservative errors compared to Ctrl mice (F_condition_ (1,36) = 9.95, *p* = 0.003). L) As percentage criteria increases, all groups require more entries to reach the criteria (F_*criteria*_(1.4,37.82) = 18.69, *p* < 0.001), APP/PS1 mice overall require more entries to reach the criteria (F_genotype_(1,27) = 17.39, *p* < 0.001) compared to WT mice and there was a significant interaction between genotype and criteria (F_*genotype* *x* *criteria*_(1.4,37.82) = 5.43, *p* = 0.01). M) The succession rate at a 90% criterion revealed a significant difference between the groups (χ^2^(3) = 26.6, *p* < 0.001) and post-hoc comparisons revealed that APP/PS1 mice that were exposed to ELS have a significantly lower RL succession rate compared to APP/PS1 mice that were exposed to Ctrl (*P* < 0.01). WT Ctrl *n* = 11, WT ELS *n* = 9, APP/PS1 Ctrl *n* = 9, APP/PS1 ELS *n* = 11. Data is presented as mean±SEM. *genotype effect *p* < 0.05, ^#^condition effect *p* < 0.05, ^@^criteria effect *p* < 0.001, ∧genotype x criteria interaction effect *p* < 0.05.

After habituation, mice had to earn their food by performing MCRL, during which a wall with 3 entrances was presented (Fig. 4A). During discrimination learning (DiL), the left entrance is rewarding. After 2 days, the rewarding entrance shifts to the right during the reversal learning trial (RL), and mice need to flexibly adjust their cognitive strategy. During DiL, APP/PS1 mice performed a higher number of total entries compared to WT mice (F_genotype_(1,36) = 6.45, *p* = 0.02) and mice that were exposed to ELS performed a higher number of total entries compared to Ctrl animals (F_condition_(1,36) = 8.24, *p* = 0.007) (Fig. 5A). Overall, all groups performed similarly during DiL in terms of percentage of correct entries (Fig. 5B). Over the course of 2 days of DiL, all groups increased the number of correct entries (F_day_(1,36) = 69.26, *p* < 0.001). APP/PS1 choose more correct entries compared to WT mice (F_genotype_(1,36) = 4.78, *p* = 0.04) and ELS mice also selected more correct entries compared to Ctrl mice (F_condition_(1,36) = 7.32, *p* = 0.01) (Fig. 5C). APP/PS1 mice displayed more incorrect entries (middle and right entries) compared to WT mice (F_genotype_(1,36) = 5.89, *p* = 0.02) (Fig. 5D). Using percentage of correct entries criteria, ranging from to 70-90% and based on a moving window of the last 30 entries, we assessed the progression of succession. During DiL, all 4 groups similarly increased the number of entries required to reach the criteria (F_criteria_(2.41,86.59) = 3.48, *p* < 0.001) (Fig. 5E). In line with these findings, an equal DiL succession rate for the individual mice, at a criterion of 90% correct entries, is visible in the Kaplan-Meier survival plot for all experimental groups (Fig. 5F). In summary, neither genotype nor condition affected DiL performance in 3-month-old mice.

Similar to DiL, APP/PS1 mice performed a higher number of total entries during reversal learning (RL) compared to WT mice (F_genotype_(1,36) = 15.59, *p* < 0.001) and mice that were exposed to ELS performed a higher number of total entries compared to Ctrl animals (F_condition_(1,36) = 9.75, *p* = 0.004) (Fig. 5G). In terms of overall percentage of correct entries, all groups performed similarly during RL (Fig. 5H). Split over the 2 days of RL, all groups increased their number of correct entries (F_day_(1,36) = 22.58, *p* < 0.001) (Fig. 5I). Additionally, APP/PS1 mice showed more correct entries compared to WT mice (F_genotype_(1,36) = 8.34, *p* = 0.007) and ELS mice choose more correct entries compared to Ctrl mice (F_condition_(1,36) = 4.66, *p* = 0.04), regardless of the day of the task. All groups decreased their number of neutral errors (i.e., middle entries) (F_day_(1,36) = 10.29, *p* = 0.003) and APP/PS1 mice displayed overall more neutral errors compared to WT mice (F_genotype_(1,36) = 8.36, *p* = 0.006) (Fig. 5J). All groups decreased their number of perseverative errors (left entries) over the 2 days of RL days (F_day_(1,36) = 233.58, *p* < 0.001) (Fig. 5K). Regardless of the 2 days, APP/PS1 mice performed more perseverative errors compared to WT mice (F_genotype_(1,36) = 8.38, *p* = 0.006) and ELS mice perform more perseverative errors compared to Ctrl mice (F_condition_(1,36) = 9.95, *p* = 0.003). During RL, all 4 groups similarly increased the number of entries required to reach the criteria over a range of 70-90% correct entries (F_criteria_(1.4,37.82) = 18.69, *p* < 0.001) (Fig. 5L). Moreover, APP/PS1 mice overall required more entries to reach the criteria (F_genotype_(1,27) = 17.39, *p* < 0.001) compared to WT mice, with a significant interaction between genotype and criterion (F_genotypexcriteria_(1.4,37.82) = 5.43, *p* = 0.01). Post hoc comparisons revealed that at higher criteria (83–90%) APP/PS1 mice required significantly more entries compared to WT mice (*p* < 0.05), whereas this was not significant at lower criteria (70–80%), suggesting that the genotype effect is most apparent at a relatively high criterion. Finally, the succession rate at the 90% criterion was assessed using a Kaplan-Meier survival plot (Fig. 5M) and revealed a significant difference between the groups (χ^2^(3) = 26.6, *p* < 0.001). Post-hoc comparisons revealed that APP/PS1 mice that were exposed to ELS have a significantly lower RL succession rate compared to APP/PS1 mice that were exposed to Ctrl (*p* < 0.01). Altogether, these findings indicate that 3-month-old APP/PS1 mice are significantly impaired in RL compared to WT mice and, at a high criterion, ELS significantly impairs RL specifically in APP/PS1 mice.

### ELS and genotype affects synaptosomal protein content in 3-month-old mice, with synaptosomal GluA1 and GluA3 content negatively correlating with RL performance

**Fig. 6:**
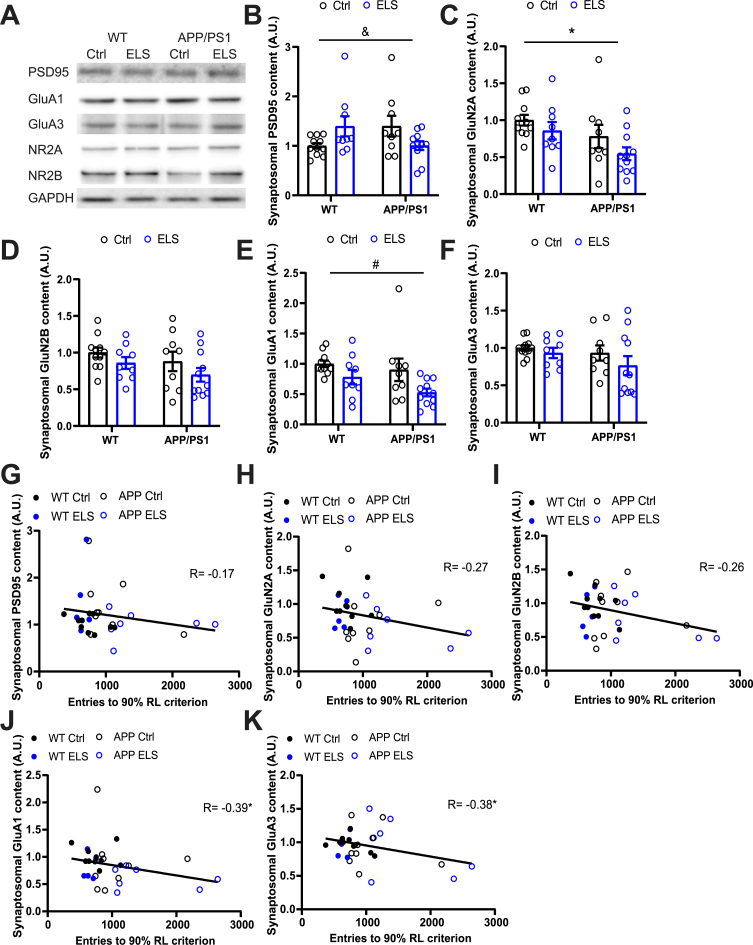
ELS and genotype affects synaptosomal protein content in 3-month-old mice and synaptosomal GluA1 and GluA3 content negatively correlate with RL performance. A) Representative bands from western blots on hippocampal synaptosomal fractions. B) ELS differentially affects synaptosomal PSD95 content depending on the genotype (F_genotype x condition_(1,36) = 7.47, *p* = 0.01), post-hoc analysis did not reveal any differences between the groups. C) APP/PS1 mice have a significantly lower synaptosomal GluN2A content compared to WT mice (F_genotype_(1,36) = 5.99, *p* = 0.02). D) No differences were observed in the synaptosomal GluN2B content between the experimental groups. E) Mice that were exposed to ELS have a significantly lower synaptosomal GluA1 content compared to Ctrl mice (F_condition_(1,36) = 6.96, *p* = 0.02). F) No differences were observed in the synaptosomal GluA3 content between the experimental groups. Ctrl WT *n* = 11, ELS WT *n* = 9, Ctrl APP/PS1 *n* = 9, ELS APP/PS1 *n* = 11. The number of entries required to reach a 90% RL criterion did not correlate with the synaptosomal content of PSD95 (G), GluN2A (H) and GluN2B (I), regardless of experimental group. J) Synaptosomal GluA1 content negatively correlated with the number of entries required to reach a 90% criterion (r(29) = –0.39, *p* = 0.02), regardless of experimental group. K) Synaptosomal GluA3 content negatively correlated with the number of entries required to reach a 90% criterion (r(29) = –0.38, *p* = 0.03). Data is presented as mean±SEM. ^&^two-way ANOVA interaction effect of genotype and condition, *p* < 0.05. *two-way ANOVA main effect of genotype (C) and significant Pearson R correlation (J and K), *p* < 0.05. ^#^two-way ANOVA main effect of condition, *p* < 0.05.

Following the MCRL task, hippocampal synaptosome fractions were isolated to assess whether ELS alters the synaptosomal composition in 3-month-old APP/PS1 mice and whether cognitive performance correlates with synaptosomal protein content. When comparing all 4 experimental groups, there was a genotype x condition interaction for synaptosomal PSD95 content (F_genotypexcondition_(1,36) = 7.47, *p* = 0.01) (Fig. 6B), as ELS appeared to increase the synaptosomal PSD95 content in WT mice while it decreased in APP/PS1 mice. Post-hoc analysis did not reveal any significant differences between the groups. APP/PS1 mice displayed a significantly lower synaptosomal GluN2A content compared to WT mice, regardless of condition (F_genotype_(1,36) = 5.99, *p* = 0.02) (Fig. 6C). Mice that were exposed to ELS had a significantly lower synaptosomal GluA1 content compared to Ctrl mice, regardless of genotype (F_condition_(1,36) = 6.96, *p* = 0.02) (Fig. 6E). There were no differences between the groups for the synaptosomal GluN2B (Fig. 6D) and GluA3 content (Fig. 6F).

To assess whether the impairments in RL performance may be related to differences in synaptic composition, we correlated the number of entries required to reach the 90% RL criterion with synaptosomal protein content. While for synaptosomal PSD95, GluN2A and GluN2B content, there were non-significant, negative correlations between RL performance and protein content (Fig. 6G-I), there were significant negative correlations between RL performance and synaptosomal GluA1 (r(29) = –0.39, *p* = 0.02) and GluA3 (r(29) = –0.38, *p* = 0.03) content (Fig. 6J, K). These correlations may be driven by the datapoints originating from APP ELS and APP Ctrl animals that displayed a high number of entries (>2000) that were required to reach the 90% criterion. Future studies will be required to further substantiate ELS-induced changes in synaptic proteins. These findings suggest that both genotype and condition affect synaptosomal protein content in particular for the PSD95, GluN2A, and GluA1 levels.

## DISCUSSION

Previous studies indicate that ELS increases the susceptibility to AD by aggravating cognitive deficits and AD pathology in 6- and 12-month-old APP/PS1 mice [7, 13, 40]. Here, we assessed the effects of ELS on APP/PS1 mice at 3 months of age, an age at which generally no Aβ pathology or cognitive impairments occur in this model yet [31, 32]. We found no evidence for an acceleration, or earlier appearance of Aβ pathology at this age after ELS, nor were differences detected in BACE1 levels or in Aβ deposition in hippocampal or prefrontal cortex. It is important to note that the 6E10 antibody primarily detects Aβ plaques and not other forms of Aβ. Thus, while the same antibody allows to also measure pre-plaque forms of Aβ, as described before [41, 42], possible changes in, e.g., soluble Aβ oligomers, which also disrupt synaptic plasticity [43], are not detected now.

We found that 3-month-old animals exposed to ELS have a smaller CR^+^ interneuron population in the DG compared to Ctrl mice, regardless of genotype. Additionally, ELS increased the synaptic strength of DG granular neurons in terms of mEPSC frequency at 3 months of age. Together, this suggests that ELS impacts both the inhibitory network and synaptic activity, thereby possibly enhancing the excitation/inhibition ratio, which has been linked to AD progression [44]. Specifically, hyperexcitability of hippocampal PV^+^ interneurons is suggested to play a causal role in the early cognitive deficits in APP/PS1 mice [42]. An increased excitability of hippocampal PV^+^ interneurons could therefore act as a compensatory mechanism for the loss of CR^+^ interneurons and it would be of interest to assess the electrophysiological properties of specifically the hippocampal interneurons in 3-month-old APP/PS1 mice that were exposed to ELS.

Synaptic activity was shown before to correlate with APP endocytosis and BACE1 activity, which in turn could increase Aβ production [45, 46]. While we did not detect effects of ELS on Aβ pathology at 3 months of age, the early enhancement in synaptic strength in our study could contribute to the ELS-induced later increase in Aβ pathology, as was shown before in 6-month-old APP/PS1 mice [13]. Extracellular Aβ levels can increase upon presynaptic vesicle release even in the absence of neuronal activity [45]. The ELS-induced increase in mEPSC frequency may potentially be due to a higher level of presynaptic vehicle release. This could increase extracellular Aβ levels progressively, which may contribute to the increase in Aβ pathology in aged APP/PS1 mice that were exposed to ELS [13]. Interestingly, paired pulse facilitation (PPF), as an index of presynaptic release probability, has been reported to be increased in the CA1 of 6-month-old APP/PS1 mice [47], while ELS was found to decrease PPF in 6-month-old WT mice [48]. To test whether ELS affects presynaptic release in the DG of relatively young APP/PS1 mice, it would therefore be of interest to test PPF in 3-month-old APP/PS1 mice that were exposed to ELS and assess the progression of Aβ pathology at different ages in future studies.

By means of the current automated home cages, we assessed whether ELS affects the general behavior and cognition of 3-month-old APP/PS1 mice. During DiL, all groups required similar numbers of entries to reach a criteria range of 70-90% correct entries and, in terms of individual DiL performance, all groups progressed similarly at the highest criterion. These findings suggest that DiL is not affected by genotype or condition at this age. A study using the same behavioral paradigm found a DiL impairment in 6–7-month-old APP/PS1 mice [38], i.e., an age at which cognitive deficits are frequently reported in this model [32, 49]. The relatively young age used in our study is possibly too early to detect DiL impairments in this model, and thus for any potential aggravation thereof by ELS.

During RL, mice had to switch their cognitive strategy and flexibly shift their behavioral preference towards the right entrance instead of the left entrance. While both genotype and condition affected the number of correct entries, as APP/PS1 mice and mice that were exposed to ELS displayed a high number of correct entries compared to the corresponding control group, these groups also displayed higher levels of perseverance during the RL period. At a 90% criterion of correct entries, ELS appeared to specifically affect the RL progression of APP/PS1 mice. Thus, 3-month-old APP/PS1 mice appear to be already impaired in RL, regardless of condition, and this impairment is even more pronounced in ELS APP/PS1 mice when the criterion is at a relatively high threshold. This is in line with previous findings, where, compared to the Ctrl APP/PS1 group, aged ELS APP/PS1 mice were also impaired in reversal learning, as measured with the Barnes maze [13]. Also in AD patients, an impaired cognitive flexibility occurs at early stages of the disease [50]. Our findings indicate that ELS shifts the early AD-associated deficits in cognitive flexibility to a relatively young age, 3 months old, in APP/PS1 mice.

Upon fractioning hippocampal synapses, we found that APP/PS1 mice have significantly lower GluN2A synaptosomal content. GluN2A is suggested to be neuroprotective [51] and its loss may therefore render the brain more susceptible to AD pathology. Regarding AMPAR content, mice that were exposed to ELS had a significantly lower synaptosomal GluA1 content compared to Ctrl mice. Given the critical role of GluA1 in synaptic plasticity, a paired decrease in synaptic content of GluA1 and GluN2A may render APP/PS1 mice more vulnerable to develop cognitive impairments.

In summary, exposure to ELS does not promote Aβ pathology in the hippocampus and prefrontal cortex of 3-month-old APP/PS1 male mice. ELS does lead, however, to a higher synaptic strength and a smaller CR^+^ interneuron population in the DG, both of WT and APP/PS1 mice. Finally, ELS and the APP/PS1 genotype do not affect DiL, while APP/PS1 mice are impaired in RL. ELS further exacerbates the RL impairment in APP/PS1 at a high RL criterion. Both condition and genotype are associated with altered postsynaptic protein content. Together, these findings may contribute to an accelerated cognitive impairment in APP/PS1 mice by ELS and potentially lead to ELS-enhanced vulnerability to AD-pathology in late adulthood. Elucidating in future studies whether and how synaptosomal protein content shapes the susceptibility and progression of AD-pathology, will provide valuable insights into AD etiology and possible preventive or therapeutic targets for the disease.

## Data Availability

The data supporting the findings of this study are available upon reasonable request.
